# Validation of influenza vaccination status using health administrative databases by integrating pharmacy claims and medical billing databases in Ontario, Canada

**DOI:** 10.1186/s12879-025-11014-1

**Published:** 2025-05-04

**Authors:** Razan Amoud, Jeffrey C. Kwong, Colleen Maxwell, Suzanne L. Tyas, Martin Cooke, Alejandro Hernandez, Wasem Alsabbagh

**Affiliations:** 1 School of Pharmacy, Faculty of Science, University of Waterloo, 10 A Victoria St. S. Kitchener, Waterloo, ON N2G 1 C5 Canada; 2 Dalla Lana School of Public Health, University of Toronto, Toronto, ON Canada; 3 Department of Family and Community Medicine, Toronto, ON Canada; 4 ICES, Toronto, ON Canada; 5 University Health Network, Toronto, ON Canada; 6 School of Public Health Sciences, Faculty of Health, University of Waterloo, Waterloo, ON Canada; 7 Department of Sociology and Legal Studies, Faculty of Arts, University of Waterloo, Waterloo, ON Canada

**Keywords:** Influenza Vaccination, Administrative Health Data, Validation, Pharmacist, Immunization

## Abstract

**Background:**

Determining vaccination status among the population is key for vaccine research and surveillance. This study aimed to validate the combined use of Ontario Health Insurance Program (OHIP) physician billing claims and Ontario Drug Benefit program (ODB) pharmacist billing claims against data from the Canadian Community Health Survey (CCHS).

**Methods:**

OHIP and ODB billing claims databases were linked to 2013–2014 CCHS data, which contain self-reported seasonal influenza vaccination status of respondents (the reference standard). Sensitivity, specificity, positive predictive value (PPV), and negative predictive value (NPV), and their associated 95% confidence intervals (CIs) were estimated. Subgroup analyses were performed based on key respondent characteristics, including having a regular medical doctor and the presence of risk factors for influenza complications.

**Results:**

There were 31,390 eligible CCHS respondents aged ≥ 12 years in Ontario who responded to the influenza vaccination questions and agreed to have their responses linked to health administrative databases. More than half (55%) were female, 29% were aged ≥ 65 years, 93% had a regular medical doctor, and 54% had one or more risk factors for influenza complications. The sensitivity for the combined administrative databases was 60.1% (95% CI, 59.3%–61.0%), specificity was 98.5% (95% CI, 98.3%–98.7%), PPV was 96.7% (95% CI, 96.3%–97.1%), and NPV was 76.9% (95% CI, 76.4%–77.5%). Sensitivity was higher among those aged ≥ 65 years (72.7%; 95% CI, 71.6%–73.7%), with a regular medical doctor (61.1%; 95% CI, 60.3%–62.0%), and those with at least one risk factor for influenza complications (65.8%; 95% CI, 64.9%–66.8%).

**Conclusion:**

Combining administrative physician and pharmacy claims data in Ontario results in moderate sensitivity but very high specificity and PPV, demonstrating that they can be a valid measure of influenza vaccination status.

**Supplementary Information:**

The online version contains supplementary material available at 10.1186/s12879-025-11014-1.

## Introduction

Influenza remains a significant public health challenge in Canada, contributing to approximately 12,200 hospitalizations and 3,500 deaths annually [1, 2]. This high burden of illness underscores the critical need for effective prevention strategies. Vaccination plays a vital role in reducing the spread and severity of influenza [3, 4]. Consequently, the Canadian province of Ontario offers influenza vaccines free of charge to all residents aged ≥ 6 months.

Accurately determining vaccination status within the population is essential for advancing vaccine research, monitoring public health activities, and evaluating the effectiveness and impact of immunization programs. To assess the impact of vaccination efforts, it is essential to accurately measure vaccine effectiveness. Studies on vaccine effectiveness rely on valid data about individuals’ vaccination status and their subsequent health outcomes [5]. These data allow researchers to evaluate how well the vaccine prevents illness, hospitalizations, and severe complications across diverse population groups. Valid measures of vaccination status are needed for vaccine effectiveness studies to produce accurate estimates, which in turn are necessary for guiding public health policies and resource allocation [6]. Moreover, understanding the effectiveness of the vaccine helps to refine future vaccination campaigns so that they continue to provide optimal protection to the population.

Vaccination status can be determined using vaccination registries, self-report questionnaires, patient medical records, or administrative databases [7]. Previous studies suggest self-report should be the reference standard for determining influenza vaccination status in the absence of registries [7–11]. However, due to the absence of influenza vaccine registries in Ontario, and challenges obtaining timely self-reported vaccination status, administrative databases become an essential resource to measure influenza vaccination status.

Until recently, physician billing claims for administering influenza vaccines, which are captured in the Ontario Health Insurance Plan (OHIP) database, were the main source for ascertaining influenza vaccination status. A previous validation study by Schwartz et al., which compared OHIP claims between 2007 and 2009 against self-reported vaccination status from the Canadian Community Health Survey (CCHS), showed that the claims data were moderately sensitive (49.8% [95% Confidence Interval (CI), 49.0%–50.5%]) and highly specific (95.7% [95% CI, 95.5%–96.0%]) in correctly identifying vaccination status. [12] The positive and negative predictive values were 88.4% (95% CI, 87.8%–89.0%) and 74.5% (95% CI, 74.0%–74.9%), respectively.

In 2012, Ontario authorized pharmacists to administer influenza vaccines in community pharmacies, which facilitated access and increased vaccine coverage in the population [13, 14]. As a result, pharmacies became the most common place for influenza vaccination in Ontario [15]. These claims are recorded in the Ontario Drug Benefit (ODB) database. Despite the growing role of pharmacies in vaccination efforts, the approach of including pharmacy claims along with OHIP physician billing claims to assess influenza vaccination status has yet to be validated in Ontario.

The objective of this study was to validate the measure of vaccination status derived from combining OHIP and ODB data in Ontario against the reference standard of self-report in the CCHS.

## Methods

### Study design & data sources

We conducted a validation study using CCHS survey data linked to OHIP, ODB, and other health administrative databases (described below). These databases were linked using unique encoded identifiers and analyzed at ICES. ICES is an independent, non-profit research institute whose status under Ontario’s health information privacy law allows it to collect and analyze health care and demographic data, without consent, for health system evaluation and improvement [16].

CCHS is a cross-sectional survey targeting a representative sample of Canadians aged ≥ 12 years across all provinces and territories, excluding those living on First Nations reserves or other Indigenous settlements, full-time members of the Canadian Armed Forces, institutionalized individuals, and children in foster care [17, 18]. Excluded individuals represent less than 3% of the population aged ≥ 12 years [19]. The annual survey cycles sample 65,000 respondents recruited by a multi-stage sampling strategy and weighted to provide valid estimates of the Canadian population [18]. The CCHS has the capability to combine two consecutive annual samples using appropriate weighting techniques, enabling researchers to increase sample size and improve the precision of estimates [18]. The CCHS uses a sampling method designed to produce representative samples at both provincial and national levels. Thus, the survey's respondents reflect the demographic and health characteristics of the broader populations in each province and across Canada.

The OHIP database captures physician billing claims for the reimbursement of clinical services provided in physician offices, including influenza vaccination [20].

Information on pharmacist billing claims for influenza vaccinations administered in community pharmacies, including the vaccine’s unique product number and service date, are recorded in the ODB database. Clinical services including the vaccine and administration fees are covered by the ODB program for all Ontario residents aged six months or older [21]. As such, all vaccinations of Ontario residents by pharmacists are captured in this database.

Demographic characteristics of respondents (age, sex) and postal code were extracted from Ontario’s Registered Persons Database (RPDB). The Postal Code Conversion File (PCCF +) dataset was used to link postal codes to Census data to derive area-based relative measures of residence for each individual (urban vs rural, with the latter defined as a community with ≤ 10,000 residents) [22]. Further, the PCCF + was also used to link each respondent’s residential postal code to Census dissemination area (population of between 400 and 700 people) median household income quintiles as a proxy for individual socioeconomic status.

Respondents’ clinical characteristics related to the risk for serious influenza infection complications (i.e., risk factors) were identified using previously validated algorithms involving diagnostic codes recorded in the Canadian Institute for Health Information (CIHI) Discharge Abstract Database (DAD), the National Ambulatory Care Reporting System (NACRS), the Same Day Surgery (SDS) database, and the OHIP database (details in Appendix 1) [23–29].

Ethics approval for this study was obtained from the University of Waterloo, Office of Research Ethics (CREB# IRB00007409, HREB# IRB00002419) in accordance with the Tri-Council Policy Statement: Ethical Conduct for Research Involving Humans (TCPS 2), Canada.

### Study sample

All CCHS respondents interviewed during the 2013 and 2014 cycles were included if they had agreed to having their data linked to administrative databases, answered the “flu shot” theme questions, and were Ontario residents with OHIP coverage for at least one year prior to the CCHS interview date. At the time this study was conducted, the most recent CCHS cycles linked and accessible at ICES were the 2013 and 2014 cycles. Respondents interviewed for both cycles were only included once during the earliest cycle (i.e., 2013). Respondents whose physician claims data for influenza vaccination indicated only an “incentive fee” billing code (Q130), used by physicians to indicate that a patient has received an influenza vaccine elsewhere, without a corresponding pharmacy or physician claim, were excluded as this was considered an ambiguous vaccination status. In a sensitivity analysis, these respondents were included as vaccinated.

### Measures

Demographic variables examined included sex, age group, rurality, and neighbourhood-level median household income quintile. Data on year of survey and having a regular medical doctor were extracted from the CCHS. A “regular medical doctor” generally refers to a family doctor or general practitioner who offers ongoing primary care and serves as the main point of contact for various health concerns, including specialist referrals. The risk groups for influenza-related complications were selected based on recommendation statements from Canada’s National Advisory Committee on Immunization (NACI). The following risk factors were identified: anemia, asthma, cancer, cardiovascular disease (including arrhythmia, hypertension, ischemic heart disease, myocardial infarction, and stroke), chronic obstructive pulmonary disease (COPD), dementia, and being immunocompromised. These risk factors guided the selection of the groups included in the analysis (Appendix 1).

#### Influenza vaccination status

The CCHS included a set of flu shot-themed questions that were asked of every respondent. We developed the following algorithm to determine the vaccination status of respondents (Appendix 2). Respondents were asked “Have you ever had a seasonal flu shot, excluding the H1 N1 flu shot?”. If the response was “NO”, respondents were considered unvaccinated. If they said “YES”, they were prompted to indicate the timing of this vaccination relative to the interview date (less than 1 year ago, 1 year to less than 2 years ago, or 2 years ago or more). Only those choosing “less than 1 year ago” for their most recent influenza vaccination were considered vaccinated. Individuals who provided ambiguous responses to either of the two questions such as “Don’t Know/Refuse [to answer]” were removed from the study sample. Previous validation studies have used a similar approach for ascertaining influenza vaccination status [12, 30, 31].

In the administrative databases, respondents’ vaccination status was determined by the presence of a billing code for influenza vaccination in OHIP or ODB (codes available in Appendix 3) [32, 33]. The timing of billing codes relative to the CCHS interview date was used to determine influenza vaccination status. The vaccination lookback window extended from the interview date to 365 days prior. Individuals vaccinated on the interview day were considered vaccinated. If multiple vaccination dates were recorded in OHIP or ODB, the closest date to the interview was used.

### Data analysis

We presented the distribution of respondents’ demographic and clinical characteristics by their CCHS vaccination status. Chi-square (χ^2^) tests for each categorical variable by exposure (vaccination status) were performed to check for bivariate statistical significance. The *p-*value for statistical significance was < 0.05. The sensitivity, specificity, positive predictive value (PPV), and negative predictive value (NPV) were calculated (with 95% CIs), assessing the validity of the combined administrative databases (OHIP and ODB) compared with the reference (CCHS) [34, 35]. Sensitivity was defined as the proportion of individuals vaccinated according to the reference standard (CCHS) who were also identified as vaccinated in the administrative data source (pharmacy claims or physician billing databases), among all individuals vaccinated in CCHS. Specificity was defined as the proportion of individuals unvaccinated in CCHS who were also classified as unvaccinated in the administrative data source, among all individuals unvaccinated in CCHS. PPV was calculated as the proportion of individuals identified as vaccinated in the administrative data source who were confirmed as vaccinated in the reference standard. NPV was the proportion of individuals classified as unvaccinated in the administrative data source who were also unvaccinated in the reference standard. The 95% confidence intervals were calculated using the Clopper-Pearson exact method.

### Subgroup analysis

Subgroup analyses were conducted by CCHS cycle year (2013 and 2014), sex, age group, rural/urban residence, presence of a regular medical doctor, and risk factors for influenza-related complications (one or more as well as individual risk factors, including arrhythmia/hypertension/ischemic heart disease/myocardial infarction/stroke), COPD, dementia, and being immunocompromised.

### Sensitivity analysis

Three sensitivity analyses were carried out. First, to determine if excluding respondents with ambiguous vaccination status—identified by the presence of the “incentive fee” code (Q130) without a corresponding ODB or OHIP claim—affected the results, we re-ran the analyses including these respondents as vaccinated. Second, since participants were asked about the 365 days before the interview, there is a risk of misclassification of the immunization year (whether it was during the most recent influenza vaccination campaign or the previous one) [12]. To minimize this risk, we conducted a sensitivity analysis by only including respondents interviewed outside influenza vaccination campaigns (between February 1 and August 31, in both 2013 and 2014), so that their answers specifically reflected the most proximal season to the date of interview. This sensitivity analysis aimed to include individuals vaccinated after the peak influenza vaccination campaign period, which typically occurs in the fall and early winter. It is important to distinguish between the timing of influenza vaccination campaigns and the influenza season, when virus circulation is highest. As this study focused on the accuracy of vaccination records rather than influenza infection risk, the relevant time period was the vaccination campaign period, when vaccines were administered, rather than the influenza season itself.Finally, since billing timestamps in administrative databases might not always match vaccine administration dates due to potential delays in billing, we conducted a sensitivity analysis allowing an extra 30 days of billing delay. Thus, we only considered vaccinations billed in 335 days before the interview date.

## Results

**Fig. 1:**
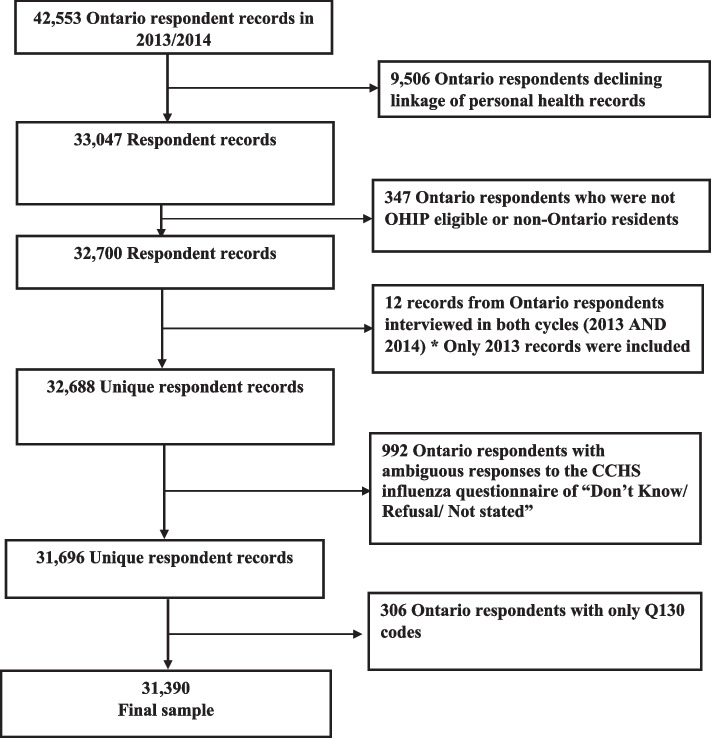
Flowchart of the study sample for Canadian Community Health Survey 2013/2014 respondents. OHIP = Ontario Health Insurance Plan, CCHS = Canadian Community Health Survey

There were 42,553 respondents aged ≥ 12 years interviewed in Ontario during CCHS cycles 2013 and 2014 [17, 18]. The number of respondents who agreed to data linkage was 33,047 (77.5%). After applying the inclusion and exclusion criteria, the final study sample included 31,390 CCHS respondents (Fig. 1).

A total of 13,358 (42.6%) of these CCHS respondents reported receiving an influenza vaccination in the past 12 months (Table 1). This was higher for those surveyed in 2014 than in 2013 (44.4% versus 40.8%, *p*-value < 0.0001), for females than for males (45.3% versus 39.2%, *p*-value < 0.0001), and for those aged ≥ 65 years than those younger than 65 (71.4% versus 30.7%, *p*-value < 0.0001). There was a similar proportion reporting having received the influenza vaccination across urban and rural settings (~ 43%, *p*-value = 0.65) and across neighbourhood income quintiles (~ 41-–43%, *p*-value = 0.1973). However, a larger proportion of individuals with a regular medical doctor had been vaccinated compared to those without one (44.2% versus 20.4%, *p*-value < 0.0001). The proportion of respondents with any risk factors for influenza-related complications who were vaccinated was 55.4%, but this percentage ranged from 78.4%, among respondents with dementia to 45.2% among respondents with asthma.

**Table 1:** Descriptive characteristics by influenza vaccination status within the previous 12 months according to the Canadian Community Health Survey

Characteristics	Respondents who received influenza vaccine, no (Row %)	Respondents who did not receive the influenza vaccine, no (Row %)	*p*-value
	***n* = 13,358 (42.6%)**	***n* = 18,032 (57.4%)**	
Canadian Community Health Survey Year of Interview			
2013	6625 (40.8%)	9604 (59.2%)	< 0.0001
2014	6733 (44.4%)	8428 (55.6%)	
Sex			
Female	7824 (45.3%)	9443 (54.7%)	< 0.0001
Male	5534 (39.2%)	8589 (60.8%)	
Age group			
12–17	694 (27.9%)	1792 (72.1%)	< 0.0001
18–49	2595 (22.8%)	8810 (77.2%)	
50–64	3559 (42.4%)	4825 (57.6%)	
65 +	6510 (71.4%)	2605 (28.6%)	
< 65	6848 (30.7%)	15,427 (69.3%)	
Residence*			
Urban	10,503 (42.5%)	14,205 (57.5%)	0.65
Rural	2851 (42.8%)	3810 (57.2%)	
Neighbourhood income quintile§			
1 (least affluent)	2509 (43.4%)	3267 (56.6%)	0.1973
2	2745 (42.6%)	3698 (57.4%)	
3	2585 (41.4%)	3654 (58.6%)	
4	2766 (42.3%)	3773 (57.7%)	
5	2723 (43.1%)	3597 (56.9%)	
Has a Regular Doctor¥			
Yes	12,919 (44.2%)	16,335 (55.8%)	< 0.0001
No	429 (20.4%)	1671 (79.6%)	
Influenza complication risk factors			
Any Risk Factor^€^	9390 (55.4%)	7554 (44.6%)	< 0.0001
Anemia	1051 (62.0%)	645 (38.0%)	< 0.0001
Arrhythmia	902 (73.6%)	324 (26.4%)	< 0.0001
Asthma	2091 (45.2%)	2531 (54.8%)	< 0.0001
Cancer	1580 (68.8%)	718 (31.2%)	< 0.0001
Cardiovascular Disease (Arrhythmia/HTN/IHD/MI/Stroke)	2520 (70.4%)	1060 (29.6%)	< 0.0001
Chronic Kidney Disease	569 (75.6%)	184 (24.4%)	< 0.0001
Congestive Heart Failure	658 (76.8%)	199 (23.2%)	< 0.0001
COPD	2142 (65.0%)	1151 (35.0%)	< 0.0001
Dementia	207 (78.4%)	57 (21.6%)	< 0.0001
Diabetes	2640 (66.5%)	1327 (33.5%)	< 0.0001
Frail	132 (75.4%)	43 (24.6%)	< 0.0001
Hypertension	6580 (65.0%)	3542 (35.0%)	< 0.0001
Immunocompromised	370 (75.5%)	120 (24.5%)	< 0.0001
Ischemic heart disease	1643 (71.2%)	665 (28.8%)	< 0.0001
Myocardial Infarction	722 (70.5%)	302 (29.5%)	< 0.0001
Stroke	403 (69.4%)	178 (30.6%)	< 0.0001

^*^ Missing data on 21 respondents

^§^ Missing data on 73 respondents

¥ Missing data ('Don't Know') on 36 respondents

€ Anemia, Arrhythmia, Asthma, Cancer, Cardiovascular Disease (Arrhythmia/Hypertension/Ischemic heart disease, Myocardial Infarction, Stroke), Chronic Kidney Disease, Congestive Heart Failure, COPD, Dementia, Diabetes, Frail, Hypertension, Immunocompromised, Ischemic heart disease, Myocardial Infarction, Stroke

There were 8,308 individuals ascertained as having received influenza vaccination in the combined (OHIP and ODB) administrative data (Table 2). Of these, 6,238 (75.1%) individuals were captured by OHIP only, 1,994 (24.0%) were captured by ODB only, and 76 (0.9%) had both physician and pharmacy billing claims. There were 274 respondents (0.87% of the total study population) identified as being vaccinated based on OHIP/ODB data who reported in the CCHS that they had not been vaccinated.

**Table 2:** Performance measures of administrative data (Ontario Health Insurance Program + Ontario Drug Benefit Program) in identifying influenza vaccination status, using the Canadian Community Health Survey as reference standard, stratified by respondent characteristics

Characteristics	True Positive	False Positive	True Negative	False Negative	Sensitivity (95% CI)	Specificity (95% CI)	PPV (95% CI)	NPV (95% CI)
Total (*n* = 31,390) Total Vaccinated in CCHS = 13,358 (43%)	8034	274	17,758	5324	60.1% (59.3%−61.0%)	98.5% (98.3%−98.7%)	96.7% (96.3%−97.1%)	76.9% (76.4%−77.5%)
Canadian Community Health Survey Year								
2013 (*n* = 16,229) Total Vaccinated in CCHS = 6,625 (41%)	3795	146	9458	2830	57.3% (56.1%−58.5%)	98.5% (98.2%−98.7%)	96.3% (95.7%−96.9%)	77.0% (76.2%−77.7%)
2014 (*n* = 15,161) Total Vaccinated in CCHS = 6,733(44%)	4239	128	8300	2494	63.0% (61.8%−64.1%)	98.5% (98.2%−98.7%)	97.1% (96.5%−97.6%)	76.9% (76.1%−77.7%)
Sex								
Female (*n* = 17,267) Total Vaccinated in CCHS = 7,824 (45%)	4728	143	9300	3096	60.4% (59.3%−61.5%)	98.5% (98.2%−98.7%)	97.1% (96.6%−97.5%)	75.0% (74.3%−75.8%)
Male (*n* = 14,123) Total Vaccinated in CCHS = 5,534 (39%)	3306	131	8458	2228	59.7% (58.4%−61.0%)	98.5% (98.2%−98.7%)	96.2% (95.5%−96.8%)	79.2% (78.4%−79.9%)
Age group								
12–17 (*n* = 2,486)) Total Vaccinated in CCHS = 694 (28%)	224	31	1761	470	32.3% (28.8%−35.9%)	98.3% (97.6%−98.8%)	87.8% (83.2%−91.6%)	78.9% (77.2%−80.6%)
18–49 (*n* = 11,405)) Total Vaccinated in CCHS = 2,595 (23%)	1121	78	8732	1474	43.2% (41.3%−45.1%)	99.1% (98.9%−99.3%)	93.5% (92.0%−94.8%)	85.6% (84.9%−86.2%)
50–64 (*n* = 8,384)) Total Vaccinated in CCHS = 3,559 (42%)	1959	68	4757	1600	55.0% (53.4%−56.7%)	98.6% (98.2%−98.9%)	96.7% (95.8%−97.4%)	74.8% (73.7%−75.9%)
< 65 (*n* = 22,275) Total Vaccinated in CCHS = 6,848 (31%)	3304	177	15,250	3544	48.3% (47.1%−49.4%)	98.9% (98.7%−99.0%)	94.9% (94.1%−95.6%)	81.1% (80.6%−81.7%)
65 + (*n* = 9,115) Total Vaccinated in CCHS = 6,510 (71%)	4730	97	2508	1780	72.7% (71.6%−73.7%)	96.3% (95.5%−97.0%)	98.0% (97.6%−98.4%)	58.5% (57.0%−60.0%)
Residence								
Urban area of residence (n = 24,708) Total Vaccinated in CCHS = 10,503 (43%)	6435	236	13,969	4068	61.3% (60.3%−62.2%)	98.3% (98.1%−98.5%)	96.5% (97.0%−96.9%)	77.5% (76.8%−78.1%)
Rural area of residence (n = 6,661) Total Vaccinated in CCHS = 2,851(43%)	1598	38	3772	1253	56.1% (54.2%−57.9%)	99.0% (98.6%−99.3%)	97.7% (96.8%−98.4%)	75.1% (73.8%−76.3%)
Has a Regular Doctor								
Yes (*n* = 29,254) Total Vaccinated in CCHS = 12,919 (44%)	7894	262	16,073	5025	61.1% (60.3%−62.0%)	98.4% (98.2%−98.6%)	96.8% (96.4%−97.2%)	76.2% (75.6%−76.8%)
No (*n* = 2,100) Total Vaccinated in CCHS = 429 (20%)	137	12	1659	292	31.9% (27.5%−36.6%)	99.3% (98.8%−99.6%)	92.0% (86.4%−95.8%)	85.0% (83.4%−86.6%)
Risk factors (comorbidities) for influenza complication								
No Risk Factor (n = 14,446) Total Vaccinated in CCHS = 3,968 (27%)	1852	107	10,371	2116	46.7% (45.1%−48.2%)	99.0% (98.8%−99.2%)	94.5% (93.4%−95.5%)	83.1% (82.4%−83.7%)
Any Risk Factor (n = 16,944) Total Vaccinated in CCHS = 9,390 (55%)	6182	167	7387	3208	65.8% (64.9%−66.8%)	97.8% (97.4%−98.1%)	97.4% (97.0%−97.8%)	69.7% (68.8%−70.6%)
Anemia (*n* = 1,696) Total Vaccinated in CCHS = 1,051 (62%)	719	17	628	332	68.4% (65.5%−71.2%)	97.4% (95.8%−98.5%)	97.7% (96.3%−98.7%)	65.4% (62.3%−68.4%)
Arrhythmia (*n* = 1,226) Total Vaccinated in CCHS = 902 (74%)	637	9	315	265	70.6% (67.5%−73.6%)	97.2% (94.8%−98.7%)	98.6% (97.4%−99.4%)	54.3% (50.2%−58.4%)
Asthma (*n* = 4,622) Total Vaccinated in CCHS = 2,091 (45%)	1271	52	2479	820	60.8% (58.7%−62.9%)	98.0% (97.3%−98.5%)	96.1% (94.9%−97.1%)	75.1% (73.6%−76.6%)
Cancer (*n* = 2,298) Total Vaccinated in CCHS = 1,580 (69%)	1112	21	697	468	70.4% (68.1%−72.6%)	97.1% (95.6%−98.2%)	98.2% (97.2%−98.9%)	59.8% (57.0%−62.7%)
Cardiovascular Disease: Arrhythmia/HTN/IHD/MI/Stroke (*n* = 3,580) Total Vaccinated in CCHS = 2,520 (70%)	1761	30	1030	759	69.9% (68.1%−71.7%)	97.2% (96.0%−98.1%)	98.3% (97.6%−98.9%)	57.6% (55.3%−59.9%)
COPD (*n* = 3,293) Total Vaccinated in CCHS = 2,142 (65%)	1470	27	1124	672	68.6% (66.6%−70.6%)	97.7% (96.6%−98.5%)	98.2% (97.4%−98.8%)	62.6% (60.3%−64.8%)
Dementia (*n* = 264) Total Vaccinated in CCHS = 207 (78%)	146	10	47	61	70.5% (63.8%−76.7%)	82.5% (70.1%−91.3%)	93.6% (88.5%−96.9%)	43.5% (34.0%−53.4%)
Hypertension (*n* = 10,122) Total Vaccinated in CCHS = 6,580 (65%)	4566	99	3443	2014	69.4% (68.3%−70.5%)	97.2% (96.6%−97.7%)	97.9% (97.4%−98.3%)	63.1% (61.8%−64.4%)
Immunocompromised (*n* = 490) Total Vaccinated in CCHS = 370 (76%)	274	6	114	96	74.1% (69.3%−78.5%)	95.0% (89.4%−98.1%)	97.9% (95.4%−99.2%)	54.3% (47.3%−61.2%)
Ischemic heart disease (*n* = 2,308) Total Vaccinated in CCHS = 1,643 (71%)	1147	19	646	496	69.8% (67.5%−72.0%)	97.1% (95.6%−98.3%)	98.37% (97.5%−99.0%)	56.6% (53.6%−59.5%)
Myocardial Infarction (*n* = 1,024) Total Vaccinated in CCHS = 722 (71%)	510	8	294	212	70.6% (67.2%−73.9%)	97.4% (94.9%−98.9%)	98.5% (97.0%−99.3%)	58.1% (53.7%−62.4%)
Stroke (*n* = 581) Total Vaccinated in CCHS = 403 (69%)	287	6	172	116	71.2% (66.5%−75.6%)	96.6% (92.8%−98.8%)	98.0% (95.6%−99.2%)	59.7% (53.8%−65.4%)

### Performance measures (sensitivity, specificity, PPV, NPV)

The sensitivity of the administrative databases in capturing individual vaccination status was 60.1% (95% CI, 59.3%–61.0%), whereas the specificity was 98.5% (95% CI, 98.3%–98.7%) (Table 2). The PPV and NPV were 96.7% (95% CI, 96.3%–97.1%) and 76.9% (95% CI, 76.4%–77.5%), respectively.

### Subgroup analyses

Performance measures varied by several respondent characteristics (Table 2). Sensitivity was higher in the 2014 CCHS interview year (63.0%, 95% CI, 61.8%–64.1%) compared with the 2013 interview year (57.3%, 95% CI, 56.1%–58.5%). It was also higher for respondents aged ≥ 65 years (72.7%, 95% CI, 71.6%–73.7%) relative to those younger than 65 years (48.3%, 95% CI, 47.1%–49.4%), and for those with a regular medical doctor (61.1%, 95% CI, 60.3%‒62.0%) compared to those without (31.9%, 95% CI, 27.5%–36.6%). Sensitivity was also higher among respondents with any medical risk factors (65.8%, 95% CI, 64.9%–66.8%) compared to those with no risk factors (46.7%, 95% CI, 45.1%–48.2%). The specificity was consistently higher than 95% across all subgroups. The only subgroup showing relatively lower specificity was that for respondents with dementia where the specificity was 82.5% (95% CI, 70.1%–91.3%).

### Sensitivity analysis

None of the three sensitivity analyses showed substantial differences compared to the original analysis. Including the 304 respondents with only the Q130"incentive fee"code as vaccinated produced results consistent with the main analyses (Appendix 4). Restricting the CCHS data to only those interviewed between February and August of each survey cycle did not substantially alter the results (Appendix 4). Lastly, excluding billings in month 12 resulted in similar performance measures, with the largest change being a 3.4% decline in sensitivity (Appendix 4).

## Discussion

In this validation study that used CCHS responses as the reference standard, the combination of physician and pharmacist billing claims had moderate sensitivity (60.1%) and high specificity (98.5%) for correctly classifying respondents’ vaccination status in Ontario. These data correctly identified vaccinated respondents an estimated 96.7% of the time (PPV) and unvaccinated respondents 76.9% of the time (NPV). Sensitivity increased from the 2013 survey to the 2014 survey and was highest among older respondents and those who reported having a regular medical doctor.

The administrative databases did not capture all vaccinated Ontario residents, resulting in a sensitivity estimate of 60.1%. This could be because many residents had received influenza vaccines in settings outside of physician offices or pharmacies, such as universities, workplaces, or public health clinics, which do not report vaccination data to administrative databases, as they do not bill the Ministry of Health directly for vaccinations. In some cases, a billing claim may not have been submitted by a physician or pharmacist (e.g., forgetting to bill) or received by the Ministry of Health (e.g., technical issues). Nonetheless, most Ontario respondents were likely captured in our sample, as pharmacies and physician offices are the most common locations for receiving influenza vaccines in the province [15]. As such, our findings suggest that administrative databases can still provide a valuable measure of influenza vaccination in Ontario.

Combining physician and pharmacy billing claims appears to have increased the validity of measured influenza vaccination status. In addition to the increased sensitivity from 2013 to 2014 observed in this study, sensitivity improved by 10.3 percentage points compared to Schwartz et al.’s study. [12] Specificity increased by 2.8 percentage points, while PPV and NPV improved by 8.3 and 2.4 percentage points, respectively [12]. This improvement in validity might reflect the increased utilization of pharmacies as preferred places for influenza vaccination, a pattern that has likely become even more common in recent years. Canada’s Seasonal Influenza Vaccination Coverage Survey suggests that the increased utilization of pharmacies as a vaccination site was not merely at the expense of physician offices; rather, it mainly came at the expense of other places like workplaces, temporary vaccine clinics, community health centres, hospitals, or retirement residences [15]. In fact, the majority of respondents to the Seasonal Influenza Vaccination Coverage Survey reported that they received their vaccine in pharmacies (52%) or doctor’s offices (17%), other places such as; temporary vaccine clinics, community health centres, workplace, hospitals, retirement residence, or other places (13%) [15]. This could be due in part to the policy change and also the accessibility of pharmacies [36]. As such, combining physician and pharmacy claims improved the accuracy of administrative databases in capturing influenza vaccination status within the Ontario population, enhancing their utility for vaccine effectiveness studies.

In the absence of vaccination registries, administrative data offer a valid alternative for tracking immunization status, making them valuable for epidemiological studies. These datasets, derived from physician and pharmacy billing claims, are directly tied to healthcare encounters, providing objective evidence of vaccinations received within formal healthcare settings [37]. Although administrative data may miss vaccinations administered outside of physician offices or pharmacies, they cover large populations comprehensively and enable researchers to conduct population-level analyses. This makes administrative data a critical tool for understanding vaccination trends, evaluating coverage rates, measuring vaccine effectiveness, and informing public health policies when centralized vaccination registries are unavailable.

The relatively higher sensitivity among certain groups within our sample, including older respondents, those with a regular medical doctor, and those with comorbidities, implies that these groups are more likely to receive influenza vaccines from their physicians and pharmacists than from other providers [15, 37]. Researchers assessing vaccine effectiveness should be aware of this variation in validity estimates when evaluating vaccine effectiveness among different population subgroups. Younger respondents and those without a regular medical doctor may be more likely to receive their vaccination from other places not captured by administrative databases such as workplaces or public health clinics. Further, these findings have important implications for studies using administrative data to measure influenza vaccination coverage or vaccine effectiveness. Specifically, relying solely on claims data may underestimate true vaccine coverage, and misclassification of vaccinated individuals as unvaccinated could bias vaccine effectiveness estimates towards the null in the absence of other biases. Integrating multiple data sources may help mitigate these limitations and improve the accuracy of vaccination status ascertainment.

### Strengths and limitations

This study is the first to rigorously assess the validity of using administrative databases that combine both physician and pharmacy claims to determine individual vaccination status in Ontario. Still, several limitations should be noted. First, while 77.5% of CCHS respondents consented to data linkage, it is uncertain if there are differences between those who consented and those who refused to have their data linked [38]. However, the proportion vaccinated for all CCHS respondents was comparable to that of respondents who consented to linkage in our sample (42.6% vs. 41.4%, respectively). Additionally, 93.1% of all CCHS respondents indicated having a regular medical doctor, which was comparable with that found in this study (93.2%). These findings, though based on only two variables, suggest minimal differences between those who consented to data linkage and those who did not. Second, the CCHS data, relying on self-report, likely also suffer from recall or social desirability bias. Only 0.87% of the total study population who reported being unvaccinated in the CCHS were identified as vaccinated in the administrative databases. On the other hand, social desirability bias might explain some instances in which vaccination is self-reported in survey data but not found in administrative databases. Further, while many questions in the CCHS have been validated and shown to be reliable, it is important to note that the question regarding vaccination status has never been validated against an official registry. This is particularly relevant because there is no centralized registry specifically for influenza vaccinations in Ontario. As such, while the CCHS data on influenza vaccination status is widely used, we acknowledge that it may not fully capture the accuracy of influenza vaccination status compared to an official registry. Finally, at the time the study was conducted, the most recent available linked CCHS interviews were cycles 2013 and 2014, just two years after pharmacist influenza vaccine administration was permitted in Ontario (October, 2012). A clear trend is not discernable from only two time points. However, the sensitivity in 2014 was observed to be higher than 2013, suggesting an improvement in performance measures for detecting influenza vaccination status in administrative databases. Further research is required for a more recent update on the validity of administrative databases in measuring influenza vaccination status.

## Conclusion

This study suggests that, after Ontario’s policy change to allow influenza vaccines to be administered at pharmacies, administrative billing data that combine pharmacy and physician billing records are valid for measuring influenza vaccination status. The results of this validation study offer crucial insights into the utility of health administrative physician and pharmacy billing claims for determining influenza vaccination status, underscoring the importance of having accurate estimates for a variety of purposes, including surveillance, public health interventions, and investigations of vaccine effectiveness.

## Supplementary Information

Supplementary Material 1.

## Data Availability

The dataset from this study is held securely in coded form at ICES. While legal data sharing agreements between ICES and data providers (e.g., healthcare organizations and government) prohibit ICES from making the dataset publicly available, access may be granted to those who meet pre-specified criteria for confidential access, available at www.ices.on.ca/DAS (email: das@ices.on.ca). The full dataset creation plan and underlying analytic code are available from the authors upon request, understanding that the computer programs may rely upon coding templates or macros that are unique to ICES and are therefore either inaccessible or may require modification.

## Abbreviations

CCHS: Canadian Community Health Survey
CI: Confidence Interval
NPV: Negative predictive value
ODB: Ontario Drug Benefit
OHIP: Ontario Health Insurance Plan
PPV: Positive predictive value

## References

[1] Schanzer DL, McGeer A, Morris K. Statistical estimates of respiratory admissions attributable to seasonal and pandemic influenza for Canada. Influenza Other Respi Viruses. 2013;7(5):799–808. 10.1111/irv.12011. 10.1111/irv.12011 PMC3796862 23122189

[2] Schanzer DL, Tam TWS, Langley JM, Winchester BT. Influenza-attributable deaths, Canada 1990–1999. Epidemiol Infect. 2007;135(7):1109–16. 10.1017/S0950268807007923. 17306052 10.1017/S0950268807007923 PMC2870678

[3] Demicheli V, Jefferson T, Ferroni E, Rivetti A, Di Pietrantonj C. Vaccines for preventing influenza in healthy adults. Cochrane Database Syst Rev. 2018;2018(2). 10.1002/14651858.CD001269.pub6 10.1002/14651858.CD001269.pub6 PMC6491184 29388196

[4] Milne A. Summary of ‘Vaccines for preventing influenza in healthy children.’ Evidence-Based Child Heal A Cochrane Rev J. 2006;1(2):523–4. 10.1002/ebch.40.

[5] Ainslie KEC, Haber M, Orenstein WA. Challenges in estimating influenza vaccine effectiveness. Expert Rev Vaccines. 2019;18(6):615–28. 10.1080/14760584.2019.1622419. 31116070 10.1080/14760584.2019.1622419 PMC6594904

[6] Bloland P, MacNeil A. Defining & assessing the quality, usability, and utilization of immunization data. BMC Public Health. 2019;19(1):380. 10.1186/s12889-019-6709-1. 30947703 10.1186/s12889-019-6709-1 PMC6450010

[7] King JP, McLean HQ, Belongia EA. Validation of self-reported influenza vaccination in the current and prior season. Influenza Other Respi Viruses. 2018;12(6):808–13. 10.1111/irv.12593. 10.1111/irv.12593 PMC6185882 30028081

[8] Irving SA, Donahue JG, Shay DK, Ellis-Coyle TL, Belongia EA. Evaluation of self-reported and registry-based influenza vaccination status in a Wisconsin cohort. Vaccine. 2009;27(47):6546–9. 10.1016/j.vaccine.2009.08.050. 19729083 10.1016/j.vaccine.2009.08.050

[9] Fishbein DB, Willis BC, Cassidy WM, et al. Determining indications for adult vaccination: patient self-assessment, medical record, or both? Vaccine. 2006;24(6):803–18. 10.1016/j.vaccine.2005.07.093. 16455167 10.1016/j.vaccine.2005.07.093

[10] Mac Donald R, Baken L, Nelson A, Nichol KL. Validation of self-report of influenza and pneumococcal vaccination status in elderly outpatients. Am J Prev Med. 1999;16(3):173–7. 10.1016/s0749-3797(98)00159-7. 10198654 10.1016/s0749-3797(98)00159-7

[11] Zimmerman RK, Raymund M, Janosky JE, Nowalk MP, Fine MJ. Sensitivity and specificity of patient self-report of influenza and pneumococcal polysaccharide vaccinations among elderly outpatients in diverse patient care strata. Vaccine. 2003;21(13):1486–91. 10.1016/S0264-410X(02)00700-4. 12615445 10.1016/s0264-410x(02)00700-4

[12] Schwartz KL, Jembere N, Campitelli MA, Buchan SA, Chung H, Kwong JC. Using physician billing claims from the Ontario Health Insurance Plan to determine individual influenza vaccination status: an updated validation study. C open. 2016;4(3):E463–70. 10.9778/cmajo.20160009. 10.9778/cmajo.20160009 PMC5047797 27730110

[13] Ontario Public Drug Programs. Frequently Asked Questions for Pharmacists – October 2012: Pharmacist Administration of Publicly Funded Influenza Vaccine and Claims Submission Using the Health Network System. https://www.health.gov.on.ca/en/pro/programs/drugs/opdp_eo/notices/eo_flu_faq_20121009.pdf.

[14] Buchan SA, Kwong JC. Trends in influenza vaccine coverage and vaccine hesitancy in Canada, 2006/07 to 2013/14: results from cross-sectional survey data. Can Med Assoc J Open. 2016;4(3):E455–62. 10.9778/cmajo.20160050. 10.9778/cmajo.20160050 PMC5143025 27975047

[15] Public Health Agency of Canada. Seasonal Influenza Vaccination Coverage in Canada, 2022–2023. Ottawa, Canada; 2023. https://www.canada.ca/en/public-health/services/immunization-vaccines/vaccination-coverage/seasonal-influenza-survey-results-2022-2023/full-report.html.

[16] ICES. Working With ICES Data. https://www.ices.on.ca/use-ices-data/working-with-ices-data/. Published 2024. Accessed October 8, 2024.

[17] Statistics Canada. Canadian Community Health Survey (CCHS) - 2013- Questionnaire. https://www23.statcan.gc.ca/imdb/p3Instr.pl?Function=getInstrumentList&Item_Id=152567&UL=1V&. Published 2013. Accessed May 17, 2023.

[18] Public Health Agency of Canada. Canadian Community Health Survey - Annual Component (CCHS)-2014. https://www23.statcan.gc.ca/imdb/p3Instr.pl?Function=getInstrumentList&Item_Id=214314&UL=1V&. Published 2014. Accessed February 8, 2024.

[19] Statistics Canada. Canadian Community Health Survey (CCHS) Annual component: User guide 2014 and 2013–2014 Microdata files. 2015. https://www.statcan.gc.ca/en/statistical-programs/document/3226_D77_T1_V2.

[20] Ontario Ministry of Health. OHIP Schedule of Benefits and fees. https://www.ontario.ca/page/ohip-schedule-benefits-and-fees#section-1. Published 2024. Accessed July 15, 2024.

[21] Government of Ontario. The flu. https://www.ontario.ca/page/flu-facts. Accessed October 10, 2024.

[22] Kralj B. Measuring, “rurality” for purposes of health-care planning: an empirical measure for Ontario. Ont Med Rev. 2000;67(9):33–52.

[23] Jaakkimainen RL, Bronskill SE, Tierney MC, et al. Identification of Physician-Diagnosed Alzheimer’s Disease and Related Dementias in Population-Based Administrative Data: A Validation Study Using Family Physicians’ Electronic Medical Records. J Alzheimers Dis. 2016;54(1):337–49. 10.3233/jad-160105. 27567819 10.3233/JAD-160105

[24] Fleet JL, Dixon SN, Shariff SZ, et al. Detecting chronic kidney disease in population-based administrative databases using an algorithm of hospital encounter and physician claim codes. BMC Nephrol. 2013;14:81. 10.1186/1471-2369-14-81. 23560464 10.1186/1471-2369-14-81 PMC3637099

[25] Hall S, Schulze K, Groome P, Mackillop W, Holowaty E. Using cancer registry data for survival studies: the example of the Ontario Cancer Registry. J Clin Epidemiol. 2006;59(1):67–76. 10.1016/j.jclinepi.2005.05.001. 16360563 10.1016/j.jclinepi.2005.05.001

[26] Gershon AS, Wang C, Guan J, Vasilevska-Ristovska J, Cicutto L, To T. Identifying Individuals with Physcian Diagnosed COPD in Health Administrative Databases. COPD J Chronic Obstr Pulm Dis. 2009;6(5):388–94. 10.1080/15412550903140865. 10.1080/15412550903140865 19863368

[27] Lee DS, Tu JV, Austin PC, et al. Effect of cardiac and noncardiac conditions on survival after defibrillator implantation. J Am Coll Cardiol. 2007;49(25):2408–15. 10.1016/j.jacc.2007.02.058. 17599603 10.1016/j.jacc.2007.02.058

[28] Hux JE, Ivis F, Flintoft V, Bica A. Diabetes in Ontario: determination of prevalence and incidence using a validated administrative data algorithm. Diabetes Care. 2002;25(3):512–6. 10.2337/diacare.25.3.512. 11874939 10.2337/diacare.25.3.512

[29] Austin PC, Daly PA, Tu JV. A multicenter study of the coding accuracy of hospital discharge administrative data for patients admitted to cardiac care units in Ontario. Am Heart J. 2002;144(2):290–6. 12177647 10.1067/mhj.2002.123839

[30] Quach S, Hamid JS, Pereira JA, et al. Influenza vaccination coverage across ethnic groups in Canada. CMAJ. 2012;184(15):1673–81. 10.1503/cmaj.111628. 22966054 10.1503/cmaj.111628 PMC3478352

[31] Wang L, Jason NX, Upshur REG. Determining use of preventive health care in Ontario: comparison of rates of 3 maneuvers in administrative and survey data. Can Fam Physician. 2009;55(2):178–179.e5. https://www.cfp.ca/content/cfp/55/2/178.full.pdf. PMC2642494 19221082

[32] Ministry of Health and Long-Term Care-Ontario. Schedule of Benefits: Physician Services Under the Health Insurance Act. 2022. https://www.health.gov.on.ca/en/pro/programs/ohip/sob/physserv/sob_master.pdf. Accessed March 4, 2022.

[33] Ministry of Health: Drugs and Devices Division. Executive Officer Notice: Administering the Flu Vaccine to Children Age 2 and older under the Universal Influenza Immunization Program 2020/21. 2020. https://www.health.gov.on.ca/en/pro/programs/drugs/opdp_eo/notices/exec_office_20201218_2.pdf.

[34] Altman DG, Bland JM. Statistics Notes: Diagnostic tests 1: sensitivity and specificity. BMJ. 1994;308(6943):1552. 10.1136/bmj.308.6943.1552. 8019315 10.1136/bmj.308.6943.1552 PMC2540489

[35] Power M, Fell G, Wright M. Principles for high-quality, high-value testing. Evid Based Med. 2013;18(1):5 LP - 10. 10.1136/eb-2012-100645 10.1136/eb-2012-100645 PMC3585491 22740357

[36] Buchan SA, Rosella LC, Finkelstein M, et al. Impact of pharmacist administration of influenza vaccines on uptake in Canada. Can Med Assoc J. 2017;189(4):E146 LP-E152. 10.1503/cmaj.151027 10.1503/cmaj.151027 PMC5266568 27503864

[37] Waite NM, Cadarette SM, Campitelli MA, Consiglio GP, Houle SKD, Kwong JC. Characteristics of patients vaccinated against influenza in physician offices versus pharmacies and predictors of vaccination location: a cross-sectional study. C open. 2019;7(2):E421–9. 10.9778/cmajo.20180189. 10.9778/cmajo.20180189 PMC6588543 31227484

[38] Harron KL, Doidge JC, Knight HE, et al. A guide to evaluating linkage quality for the analysis of linked data. Int J Epidemiol. 2017;46(5):1699–710. 10.1093/ije/dyx177. 29025131 10.1093/ije/dyx177 PMC5837697

