# Supplementary material for: Validation of influenza vaccination status using health administrative databases by integrating pharmacy claims and medical billing databases in Ontario, Canada
Source: BMC Infect Dis. 2025 May 4;25:653. doi: 10.1186/s12879-025-11014-1 (PMC12051348; doi:10.1186/s12879-025-11014-1)
Supplement: Supplementary file 1 — Supplementary Material 1. [file 12879_2025_11014_MOESM1_ESM.docx]

**Appendix 1 Definitions of medical conditions increasing risk of influenza-related complications**^1^

| Medical Condition | Definition |
| --- | --- |
| Asthma | Asthma database was used to identify patients with asthma, based on 2 or more ambulatory care visits and/or 1 or more hospitalizations.^2^  **OHIP**  OHIP diagnostic code: 493  **CIHI-DAD**  ICD-9 diagnostic code: 493  ICD-10 diagnostic codes: J45, J46 |
| Chronic obstructive pulmonary disease (COPD) | COPD database was used to identify patients with COPD, based on 1 or more ambulatory care visits and/or 1 or more hospitalizations. Algorithm to identify COPD patients were only validated in those ages 35 and over.^3^  **OHIP**  OHIP diagnostic codes: 491, 492, 496  **CIHI-DAD**  ICD-9 diagnostic codes: 491, 492, 496  ICD-10 diagnostic codes: J41, J42, J43, J44 |
| Anemia | Patients with a diagnosis of anemia were identified if they had any of the following codes in OHIP, CIHI-DAD, CIHI-SDS or CIHI-NACRS in the 3 years prior to index date:  **OHIP**  OHIP diagnostic codes: 280, 281, 282, 283, 284, 285, 773  **CIHI-DAD, CIHI-SDS, CIHI NACRS**  ICD-10 diagnostic codes: D50, D51, D52, D53, D55, D56, D572, D573, D574, D58, D59, D60, D61. D63, P55, P560, P570 |
| Cancer | **OCR** was used to identify patients with any cancer diagnosed in Ontario except for non-melanoma skin cancer. ^4^ |
| Immunocompromised | Those identified using the following databases and definitions:  **ODB**  30 days of oral corticosteroids in the past 6 months, antineoplastic use in the past 6 months, or use of another immunocompromising drug in the past 6 months  **CORRLINK**  CORRLINK is a dataset in ICES which links CORR and CIHI-DAD data. This database only includes patients that have received an organ transplant and does not include dialysis patients.  **HIV**  HIV database was used to identify patients with HIV, based on 3 physician claims in 3 years with OHIP diagnostic codes: 042, 043 or 044 ^5^ |
| Dementia | **Dementia:**  1 hospitalization for dementia and/or 3 ambulatory visits for dementia, each separated by at least 30 days, within 2 years  and/or 1 prescription from ODB ^6^  **OHIP**  OHIP diagnostic codes: 290, 331  **CIHI-DAD, CIHI-SDS**  ICD-9 diagnostic codes: 0461, 290.0, 290.1, 290.2, 290.3, 290.4, 294, 331.0, 331.1, 331.5  ICD-10 diagnostic codes: F00, F01, F02, F03, G30  **ODB**  1 prescription for a cholinesterase inhibitor |
| History of congestive heart failure (CHF) | CHF database was used to identify patients with CHF, based on 1 CIHI NACRS, CIHI-DAD, CIHI-SDS, or OHIP claim and a second claim (from either) in 1 year. The CHF database is limited to those 40 years of age or older.^7^  **OHIP**  OHIP diagnostic code: 428  **CIHI-DAD, CIHI-SDS**  ICD-9 diagnostic code: 428  ICD-10 diagnostic codes: I500, I501, I509 |
| Acute Ischemic Stroke | **Acute Ischemic Stroke** ^8^**:**  CIHI-DAD was used to identify patients with a history of acute ischemic stroke, based on at least 1 hospitalization with a main diagnosis coded with one of the following codes:  ICD-9 diagnostic codes: 434, 436  ICD-10 diagnostic codes: I63 (excluding I63.6), I64, H34.1 |
| History of cardiac ischemia | CIHI-DAD and CIHI-NACRS were used to identify patients with a history of cardiac ischemia, based on at least 1 hospitalization or ED visit with a diagnosis or procedure coded with one of the following codes:  **Chronic Ischemic Heart Disease:**  ICD-9 diagnostic codes: 412, 4140, 4141, 4148, 4149, 4292  ICD-10 diagnostic codes: I25  **Myocardial infarction:**  ICD-9 diagnostic codes: 410  ICD-10 diagnostic codes: I21, I22 |
| History of cardiac arrhythmia | CIHI-DAD and CIHI-NACRS were used to identify patients with a history of cardiac arrhythmia, based on at least 1 hospitalization or ED visit with a diagnosis or procedure coded with one of the following codes:  **Atrial Fibrillation/Atrial Flutter:**  ICD-9 diagnostic codes: 4273  ICD-10 diagnostic codes: I48  **Ventricular Arrhythmia & Tachycardia:**  ICD-9 diagnostic codes: 4271, 4274, 4276, 4278  ICD-10 diagnostic codes: I470, I472, I490, I493  **Permanent Pacemaker** ^9,10^**:**  CCI procedure codes: 1HZ53GRNM, 1HZ53LANM, 1HZ53GRNK, 1HZ53LANK, 1HZ53GRNL, 1HZ53LANL  CCP procedure code: 4971  **Implantable Cardioverter- Defibrillator** ^11^**:**  CCI procedure codes: 1HZ53GRFS, 1HZ53LAFS, 1HZ53SYFS, 1HZ53HAFS  CCP procedure codes: 4974 |
| Hypertension | **Adults (20+):**^12^  - ≥1 Hospitalization *or* ≥2 OHIP in a two-year period. *OR*  - 1 OHIP *followed by* OHIP/Hosp within two years. *OR*  - 1 Hospitalization (<1991) *followed by* 1 Hospitalization (>1991) |

OHIP=Ontario Health Insurance Plan, CIHI=Canadian Institute of Health, DAD=Discharge Abstract Database, NACRS=National Ambulatory Care Reporting System, SDS=Same Day Surgery, CCI=Canadian Classification of Health Interventions, CCP=Canadian Classification of Procedures, CORR=Canadian Organ Replacement Register, ORRS=Ontario Renal Reporting System, ODD=Ontario Diabetes Database, OCR=Ontario Cancer Registry, CHF=Ontario Congestive Heart Failure Database, ODB=Ontario Drug Benefit, HIV=Ontario HIV database

**Identification of frailty in this study**^13^

**Seniors are considered frail (flagged as ‘yes’ for the binary measure of frailty) if they meet at least TWO or more of the following domains from 3a to 3g:**

3a. COGNITIVE IMPAIRMENT

| Definition | ICD9/10 | | Notes |
| --- | --- | --- | --- |
| DISCHARGE ABSTRACTS (DAD) AND/OR PHYSICIAN BILLINGS/CLAIMS DXCODES1-3 | | | |
| Senile dementia, uncomplicated | ICD9  ICD10 | ‘2900’<=DXCODE [i] <=‘2909’  ‘F0390’<= DXCODE [i] <=‘F0390’ |  |
| Dementia in Alzheimer's disease | ICD9  ICD10 | ‘3310’<=DXCODE [i]<=’3310’  ‘G300’<=DXCODE [i]<=’G300’ |  |
| Vascular dementia | ICD9  ICD10 | ‘2904’<=DXCODE [i] <=‘2904’  ‘F010’<=DXCODE [i]<=’F019’ | Captured above |
| Dementia in other diseases classified elsewhere | ICD9  ICD10 | ‘2941’<=DXCODE [i] <=’2941’  ‘F020’<=DXCODE [i]<=’F028’  ‘F03’<=DXCODE [i]<=’F03’ | Captured above |
| Unspecified dementia | ICD9  ICD10 | ‘2942’<=DXCODE [i]<=’2942’  ‘F0390’<=DXCODE [i]<=’F0391’ |  |
| Cerebral generations usually manifest in childhood | ICD9  ICD10 | ‘3300’<=DXCODE [i]<=’3309’  ‘G94’<=DXCODE [i]<=’G94’ |  |
| Other cerebral degenerations including Alzheimer’s | ICD9  ICD10 | ‘3311’<=DXCODE [i]<=’3362’  ‘G310’<=DXCODE [i]<=’G328’ |  |
| Senility without mention of psychosis | ICD9  ICD10 | ‘797’<=DXCODE [i]<=’797’  ‘R54’<=DXCODE [i]<=’R54’ |  |
| Delirium | ICD9  ICD10 | ‘293’<=DXCODE [i]<=’293’  ‘F05’<=DXCODE [i]<=’F05’ |  |

3b. GENERAL HEALTH STATUS INDICATORS

| Definition | Fields/ICD9/10 | Data file | Notes |
| --- | --- | --- | --- |
| Record of at least one of the following i to iv … | | | |
| i. At least 2 hospital admissions in past year | Admission dates | DAD | Transfers are a single admission |
| OR | | | |
| ii. At least 2 ED visits in past year | NACRS: Entry; E=emergency; addate;  registration date: location and/or hospital unit (=ED) | NACRS  Physician billings | The same methods will be used as for the ED quality indicator |
| OR | | | |
| iii. Malaise and fatigue /debility | ICD9: 7807; 7993  ICD10: R53; G933 | DAD  Physician diagnostic codes |  |
| OR | | | |
| iv. Cachexia | ICD9: 7994  ICD10: R64 | DAD  Physician diagnostic codes |  |

3c. INCONTINENCE

| Definition | ICD9/10 | | Notes |
| --- | --- | --- | --- |
| DISCHARGE ABSTRACTS (DAD) AND/OR PHYSICIAN BILLINGS/CLAIMS DXCODES1-3 | | | |
| Urinary | ICD9  ICD10 | 7883  R32 |  |
| Fecal | ICD9  ICD10 | 7876  R15 |  |

3d. FALLS

| Definition | ICD9/10 | | Notes |
| --- | --- | --- | --- |
| DISCHARGE ABSTRACTS (DAD) DXCODES1-3 | | | |
| Various falls | ICD9  ICD10 | E88, E9177, E9178, E9293  W01, W05, W06-W19 | Only count if associated with hospitalization. |

3e. NUTRITION ISSUES

| Definition | ICD9/10 | | Notes |
| --- | --- | --- | --- |
| DISCHARGE ABSTRACTS (DAD) AND/OR PHYSICIAN BILLINGS/CLAIMS DXCODES1-3 | | | |
| Record of at least one of the following i to ii… | | | |
| i. Abnormal weight loss; underweight; other concerns | ICD9  ICD10 | 7830, 78321, 78322, 7833, 7839  R630, R633, R634, R636, R638 |  |
| OR | | | |
| ii. Failure to thrive (adult) | ICD9  ICD10 | 7837  R627 |  |

3f. FUNCTIONAL PERFORMANCE

| Definition | ICD9/10 | | Notes |
| --- | --- | --- | --- |
| DISCHARGE ABSTRACTS (DAD) AND/OR PHYSICIAN BILLINGS/CLAIMS DXCODES1-3 | | | |
| Record of at least one of the following i to v… | | | |
| i. Abnormality of gait | ICD9  ICD10 | 7812  R26 |  |
| OR | | | |
| ii. Difficulty in walking | ICD9  ICD10 | 7197  R262 |  |
| OR | | | |
| iii. Muscular wasting and disuse atrophy | ICD9  ICD10 | 7282  M6250 |  |
| OR | | | |
| iv. Muscular weakness | ICD9  ICD10 | 72887  M6281 |  |
| OR | | | |
| v. Pressure ulcer | ICD9  ICD10 | 70700 - 70709  L89 |  |

3g. TARGETED HEALTH SERVICE UTILIZATION

| Definition | Fields | Data file | Notes |
| --- | --- | --- | --- |
| Record of at least one of the following i to iii … | | | |
| i. At least one geriatrician billing claim | Provider type  (spec, dspecial, doctype)  = Geriatric medicine (GEMD) | Physician billing |  |
| OR | | | |
| ii. At least one geriatrician service claim | Main patient service (patserv; mpserv)  72= geriatrics | DAD |  |
| OR | | | |
| iii. At least one provider home visit (all types) | Location of service (location, servloc)  = Patients home; home hospital care | Physician billings | Provinces may offer a number of categories. We are interested in the ‘home’ (not a nursing home which is used for decision rule #1) |

**Appendix 2 CCHS’s core-content questions about receiving the influenza vaccine.**

***FLU_160*:** Have you ever had a seasonal flu shot, excluding the H1N1 flu shot?

1: Yes 2: No 8: RF (refuse) 9: DK (Don’t Know)

***FLU_162*:** When did you have your last seasonal flu shot?

1: Less than 1 year ago

2: 1 year to less than 2 years ago

3: 2 years ago or more

8: RF

9: DK

**The algorithm of questions used in the CCHS survey on taking the flu shot.**


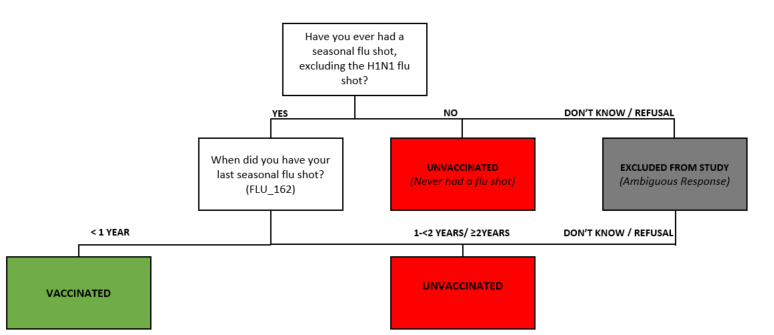


**Appendix 3 Influenza Vaccination Administrative Codes**

| **Available fee codes during years 2010-2016** | |
| --- | --- |
| **Ontario Health Insurance Program (Variable: FEECODE):** | G590 |
|  | G591 |
|  | Q590 |
|  | Q690 |
|  | Q691 |
|  | G592 |

| **Drug Identification Numbers in Ontario Drug Benefit program publicly funded as of 2012 in Ontario** | | | | |
| --- | --- | --- | --- | --- |
| **Drug Identification Number** | **Influenza Product** | **Eligibility** | **2012-13** | **2013-14** |
| 2015986 | FLUVIRAL® GlaxoSmithKline Inc. |  | x | x |
| 2223929 | VAXIGRIP® Sanofi Pasteur Limited |  | x | x |
| 2346850 | AGRIFLU® Novartis Vaccines and Diagnostics, Inc. |  | x | x |
| 2362384 | FLUAD® Novartis Vaccines and Diagnostics, Inc. (Trivalent influenza vaccine) | 65 years and older | x |  |

**Appendix 4 Sensitivity Analysis of Performance Measures after including respondents with only Q130 fee-codes in Administrative Data as vaccinated**

| **Characteristics** | **True Positive** | **False Positive** | **True Negative** | **False Negative** | **Sensitivity (95%CI,)** | **Specificity (95%CI,)** | **PPV (95%CI,)** | **NPV (95%CI,)** |
| --- | --- | --- | --- | --- | --- | --- | --- | --- |
| **Main analysis** | 8034 | 274 | 17758 | 5324 | 60.1% (59.3%-61.0%) | 98.48% (98.3%-98.7%) | 96.7% (96.3%-97.1%) | 77.0% (76.4%-77.5%) |
| **Sensitivity analysis by adding respondents with only Q130 fee code** | 8302 | 310 | 17758 | 5324 | 60.9% (60.1%-61.8%) | 98.28% (98.1%-98.5%) | 96.4% (96.0%-96.8%) | 76.9% (76.4%-77.5%) |

**Appendix 5 Performance Measures of Administrative Data (OHIP+ODB) in Identifying Influenza Vaccination Status, using CCHS as Reference standard (Restricted to CCHS Interviews from Feb. 1st to Aug. 30th)**

| **Characteristics** | **True Positive** | **False Positive** | **True Negative** | **False Negative** | **Sensitivity (95%CI,)** | **Specificity (95%CI,)** | **PPV (95%CI,)** | **NPV (95%CI,)** |
| --- | --- | --- | --- | --- | --- | --- | --- | --- |
| Main analysis | 8034 | 274 | 17758 | 5324 | 60.1% (59.3%-61.0%) | 98.5% (98.3%-98.7%) | 96.7% (96.3%-97.1%) | 77.0% (76.4%-77.5%) |
| Study Sample of sensitivity analysis by restriction  (n)= 16,651 | 4208 | 88 | 9545 | 2810 | 60.0% (58.8%-61.1%) | 99.1% (98.9%-99.3%) | 98.0% (97.5%-98.4%) | 77.3% (76.5%-78.0%) |

**Appendix 6 Sensitivity Analysis of vaccination window restriction in the administrative billing claims to up to 335 days,**

| **Characteristics** | **True Positive** | **False Positive** | **True Negative** | **False Negative** | **Sensitivity (95%CI,)** | **Specificity (95%CI,)** | **PPV (95%CI,)** | **NPV (95%CI,)** |
| --- | --- | --- | --- | --- | --- | --- | --- | --- |
| **Main analysis** | **8034** | **274** | **17758** | **5324** | **60.1% (59.3%-61.0%)** | **98.5% (98.3%-98.7%)** | **96.7% (96.3%-97.1%)** | **77.0% (76.4%-77.5%)** |
| **Sensitivity analysis** | 7607 | 211 | 17821 | 5751 | 56.7% (56.1% - 57.8%) | 98.8% (98.7% - 99%) | 97.3% (96.9% - 97.7%) | 75.6% (75.1% - 76.2%) |

**References**

1. Kwong JC, Buchan SA, Chung H, et al. Can routinely collected laboratory and health administrative data be used to assess influenza vaccine effectiveness? *Vaccine*. 2019;37(31 (suppl1):S3-S4). doi:10.1016/j.vaccine.2019.06.011

2. Gershon AS, Wang C, Guan J, Vasilevska-Ristovska J, Cicutto L, To T. Identifying patients with physician-diagnosed asthma in health administrative databases. *Can Respir J*. 2009;16(6):183-188. doi:10.1155/2009/963098

3. Gershon AS, Wang C, Guan J, Vasilevska-Ristovska J, Cicutto L, To T. Identifying Individuals with Physcian Diagnosed COPD in Health Administrative Databases. *COPD J Chronic Obstr Pulm Dis*. 2009;6(5):388-394. doi:10.1080/15412550903140865

4. Hall S, Schulze K, Groome P, Mackillop W, Holowaty E. Using cancer registry data for survival studies: the example of the Ontario Cancer Registry. *J Clin Epidemiol*. 2006;59(1):67-76. doi:10.1016/j.jclinepi.2005.05.001

5. Antoniou T, Zagorski B, Loutfy MR, Strike C, Glazier RH. Validation of Case-Finding Algorithms Derived from Administrative Data for Identifying Adults Living with Human Immunodeficiency Virus Infection. Thiem U, ed. *PLoS One*. 2011;6(6):e21748. doi:10.1371/journal.pone.0021748

6. Jaakkimainen RL, Bronskill SE, Tierney MC, et al. Identification of Physician-Diagnosed Alzheimer’s Disease and Related Dementias in Population-Based Administrative Data: A Validation Study Using Family Physicians’ Electronic Medical Records. *J Alzheimers Dis*. 2016;54(1):337-349. doi:10.3233/jad-160105

7. Schultz SE, Rothwell DM, Chen Z, Tu K. Identifying cases of congestive heart failure from administrative data: a validation study using primary care patient records. *Chronic Dis Inj Can*. 2013;33(3):160-166.

8. Lindsay M, Gubitz G, Bayley M, Hill M, Davies-Schinkel, C Singh S, Phillips S. *Canadian Best Practice Recommendations for Stroke Care (Update 2010). On Behalf of the Canadian Stroke Strategy Best Practices and Standards Writing Group.* Ottawa, Ontario Canada; 2010. https://www.gbhn.ca/ebc/documents/CBPRecommendationsforStrokeCareUpdate2010.pdf.

9. Gill SS, Anderson GM, Fischer HD, et al. Syncope and Its Consequences in Patients With Dementia Receiving Cholinesterase Inhibitors: A Population-Based Cohort Study. *Arch Intern Med*. 2009;169(9):867-873. doi:10.1001/archinternmed.2009.43

10. Juurlink D, Preyra C, Croxford R, et al. Canadian institute for health information discharge abstract database: a validation study. *ICES Investig report Inst Clin Eval Sci Toronto*. 2006.

11. Lee DS, Tu J V, Austin PC, et al. Effect of cardiac and noncardiac conditions on survival after defibrillator implantation. *J Am Coll Cardiol*. 2007;49(25):2408-2415. doi:10.1016/j.jacc.2007.02.058

12. Tu K, Campbell NR, Chen Z-L, Cauch-Dudek KJ, McAlister FA. Accuracy of administrative databases in identifying patients with hypertension. *Open Med*. 2007;1(1):e18-26.

13. Urquhart R, Giguere AMC, Lawson B, et al. Rules to Identify Persons with Frailty in Administrative Health Databases. *Can J Aging*. 2017;36(4):514-521. doi:10.1017/S0714980817000393
